# Preliminary validity and reliability of a Thai Berlin questionnaire in stroke patients

**DOI:** 10.1186/1756-0500-7-348

**Published:** 2014-06-09

**Authors:** Jittima Saengsuwan, Niramon Ungtrakul, Jiamjit Saengsuwan, Kittisak Sawanyawisuth

**Affiliations:** 1 Department of Physical Medicine and Rehabilitation, Faculty of Medicine, Khon Kaen University, Khon Kaen, Thailand; 2 North-eastern Stroke Research Group, Khon Kaen University, Khon Kaen, Thailand; 3 Center of Stroke research Berlin, Charité - Universitätsmedizin Berlin, Berlin, Germany; 4 Rehabilitation Department, Roi Et hospital, Roi Et, Thailand; 5 Faculty of Public Health, Khon Kaen University, Mittraphap Road, Khon Kaen, Thailand; 6 Department of Medicine, Faculty of Medicine, Khon Kaen University, Khon Kaen, Thailand; 7 The Research and Training Center for Enhancing Quality of Life of Working-Age People, Khon Kaen University, Khon Kaen, Thailand

**Keywords:** Stroke, Obstructive sleep apnea, Berlin questionnaire, Epworth sleepiness scale, Validity

## Abstract

**Background:**

Obstructive sleep apnea (OSA) is a major risk factor for stroke. The Berlin Questionnaire (BQ) has been shown to be a valid tool to screen for OSA. The literature has limited data on using the BQ in stroke patients; particularly in Thailand and other developing countries. Here, we aimed to develop a Thai-language Berlin Questionnaire (Thai BQ) and to preliminarily assess construct validity, test-retest reliability and the agreement of the Thai BQ with the Thai Epworth Sleepiness Scale (Thai ESS), another screening tool for OSA.

**Methods:**

A hospital-based cross-sectional study was performed from January to July, 2011. One hundred first-ever stroke patients, including acute and chronic cases, and their caregivers were enrolled. The Thai BQ was developed using the forward-backward translation method. Evaluation of construct validity was done by factor analysis. Internal consistency of the Thai BQ and the Thai ESS were evaluated using Cronbach’s alpha coefficient. Test-retest reliability and the agreement of the Thai BQ and the Thai ESS were evaluated using Cohen’s kappa coefficient.

**Results:**

Factor analysis identified 4 main factors: Factor 1–Snoring behaviour; Factor 2–Sleepiness during driving; Factor 3–Daytime fatigue; and Factor 4–Hypertension or obesity. Cronbach’s alpha coefficient was 0.77 (95% confidence interval (CI) = 0.69-0.83) and Cohen’s kappa coefficient was 0.86 (95% CI = 0.74-0.98) in the Thai BQ. Cronbach’s alpha coefficient was 0.59 (95% CI = 0.45-0.70) and the Cohen’s kappa coefficient was 0.81 (95% CI = 0.60-1.00) in the Thai ESS. The agreement between the Thai ESS and the Thai BQ was fair.

**Conclusions:**

The Thai BQ is a valid and reliable tool to screen for OSA in stroke patients. As factor analysis revealed 4 factors in contrast to the 3 factors in the original BQ, further modification of the Thai BQ is required.

## Background

The prevalence of obstructive sleep apnea (OSA) in stroke patients is higher than in the general population (55-71% vs. 12–18.5%) [1-4]. OSA is not only an independent risk factor for stroke: stroke patients with OSA also have worse outcomes, higher rates of recurrent stroke and higher mortality rates [5-7]. Moreover, recent evidence has shown that continuous positive airway pressure (CPAP) treatment in acute stroke improved neurological outcome compared to control treatment if OSA was detected [8]. Thus, it is important to identify OSA in stroke patients. OSA in stroke patients, however, has not been widely evaluated, partly because of the lack of awareness of the impact of OSA in stroke and the limited availability of polysomnography.

In an attempt to address this need, many questionnaires have been developed to identify people at risk of OSA. Two of the most widely used self-administered screening tools are the Berlin Questionnaire (BQ) and the Epworth Sleepiness Scale (ESS) [9,10]. The ESS contains 8 subjective and self-administered items relating to daytime sleepiness. In Thailand, the Thai ESS has already been translated and tested in both healthy volunteers and able-bodied participants with OSA. It has high internal consistency demonstrated by Cronbach’s alpha coefficient of 0.87 and high test-retest reliability as shown by an intra-class correlation coefficient of 0.79 [11]. For ESS, however, Onen et al. found that almost 60% of people aged more than 65 years were not able to answer at least one question in the ESS. Further, age and cognitive status were related to differences in the ESS score [12]. Many stroke patients may also have these limitations.

The BQ has a total of 11 questions in 3 categories: snoring behaviour, waketime fatigue or sleepiness, and the diagnosis of hypertension or obesity. It has good internal consistency as demonstrated by a Cronbach’s alpha value of 0.86-0.92 [9]. In addition, Ramachandran and Josephs published findings from a meta-analysis of clinical screening tests for OSA and concluded that the BQ was the most accurate questionnaire for predicting diagnosis of OSA [13].

The BQ and ESS as screening tools for OSA in stroke patients are limited in clinical practice particularly in Thailand and other developing countries (Additional file 1). Both tools are helpful for clinicians to select appropriate cases for referral to sleep facilities which are lacking in the developing world. The aims of this study were to 1) develop a Thai-language version of the BQ, 2) study the preliminary validity and reliability of the Thai BQ, and 3) compare the agreement of the caregiver-administered Thai BQ with the patient-administered Thai ESS.

## Methods

The cross-sectional study was conducted in both the in and out-patient units in Srinagarind Hospital, Thailand, from January to July 2011. The study was reviewed and approved by the Khon Kaen University Ethics Committee for Human Research (HE531422). All participants signed a consent form prior to participation.

### Subjects

The eligible population was comprised of patients older than 18 years with first-ever haemorrhagic or ischemic stroke, with radiographic evidence of cerebrovascular disease. Exclusion criteria were: 1) Mini-Mental State Examination of less than 24, aphasia or psychiatric disorder; 2) concurrent cardiopulmonary disease affecting breathing or tiredness, e.g., congestive heart failure or asthma; 3) coexisting neurological conditions such as myasthenia gravis and 4) taking medication affecting sleep.

### Translation of the original BQ into the Thai language

Translation of the BQ was kindly permitted by the original developer [9]. The translation involved converting the questions and rating criteria of the original BQ (Additional files 2 and 3). The Thai version was then translated back to English (forward-backward translation method) using two bilingual translators (one physician of rehabilitation medicine and one of sleep medicine).

The translated version was reviewed by an expert panel of four to evaluate the equivalence to the original version and the content validity. The expert panel consisted of one physician of rehabilitation medicine, one otolaryngologist, one sleep medicine physician and one sleep lab nurse. The content validity of the Thai BQ was judged by item-objective congruence (IOC). Each expert independently rated the relevance of each item on the Thai BQ and the original BQ to determine whether the translated items fell within the content domain as specified by the original BQ, using a scale of +1 (clear agreement), 0 (unclear) or −1 (no clear agreement). The IOC for each item was the summation of scores given by the experts divided by the number of experts. The average IOC score was 0.86. Every item in the Thai BQ scored more than 0.75 which is considered acceptable [14].

The Thai BQ version was then used to test structural validity, test-retest reliability and the agreement of the Thai BQ and the Thai ESS by interviewing the caregiver of stroke patients for the Thai BQ and the stroke patients themselves for the Thai ESS. All patients and caregivers completed the questionnaire twice within 48 hours for the test-retest reliability analysis. High risk patients were defined as those positive in more than 2 categories of the BQ or having a Thai ESS score equal to or more than 10.

Baseline characteristics of patients who participated were recorded: age, gender, weight, height, side of stroke and type of stroke together with underlying disease profile.

### Statistical analysis

Construct validity was tested using exploratory factor analysis to identify the latent factors underlying the 11 questions of the BQ. The internal consistency of each category was calculated using Cronbach’s alpha coefficient. The test-retest reliability of the Thai BQ and agreement between the Thai BQ and the Thai ESS were examined using Cohen’s kappa coefficient. The statistical analysis was carried out using SPSS version 19.

## Results

A total of 100 first-ever stroke patients were included in the study. Of those, fifty-one patients were male (51%). The mean age and BMI of all patients were 64 years (range 37–95 years) and 24 kg/m^2^ (Table 1). Regarding stroke types, 70 patients had ischemic strokes, while 25 patients had hemorrhagic stroke and 5 patients had both ischemic and haemorrhagic stroke. The median time after stroke was 14 days (1^st^ and 3^rd^ quartile: 3–150 days). The main comorbidities were hypertension (42%) and diabetes mellitus (34%).

**Table 1:** General characteristics of stroke patients (n = 100) who participated in the study

**Characteristics**	**Data**
Age (Years): Mean (SD)	64 (10.8)
Gender, (n)	
Male	51
Female	49
Type of stroke (n)	
Ischemic	70
Hemorrhagic	25
Mixed	5
Duration after stroke (days)	
Median	14
1^st^ quartile	3
3^rd^ quartile	150
Underlying disease (n)	
Hypertension	42
Diabetes mellitus	34
Dyslipidemia	20

Snoring and witnessed apnea was found in 67% and 3% of patients. 45% and 4% of patients were positive for categories 1 and 2 (Table 2). Twelve patients had daytime fatigue and seven patients had fallen asleep while driving. The percentage of patients with hypertension or obesity was 47%. Twenty-four patients had a high risk for OSA as identified by the Thai BQ. The average Thai ESS score of all patients was 4.6. Eight patients were at risk for OSA as identified by the Thai ESS.

**Table 2:** Numbers of responses for each item of the Thai BQ (n = 100)

**Questions**	**N**
*Category 1*	
1. Do you snore?	
Yes	67
2. Your snoring is	
Louder than talking	8
Very loud–can be heard in adjacent room	8
3. How often do you snore?	
Nearly every day	32
3-4 times a week	10
4. Has your snoring ever bother other people?	
Yes	15
5. Has anyone noticed that you quit breathing during your sleep?	
Nearly every day	2
3-4 times a week	1
*Category 2*	
6. How often do you feel tired or fatigued after your sleep?	
Nearly every day	3
3-4 times a week	3
7. During your waking time, do you feel tired, fatigue or not up to par?	
Nearly every day	7
3-4 times a week	5
8. Have you ever nodded off or fallen asleep while driving a vehicle?	
Yes	7
9. How often does this occur?	
Nearly every day	0
*Category 3*	
10. Do you have high blood pressure?	
Yes	42
11. Do you have BMI ≥ 30 kg/m^2^?	
Yes	7

### Construct validity

Principal component analysis (PCA) was conducted on the 11 items of the BQ with orthogonal rotation (varimax). The Kaiser-Meyer-Olkin measure verified the sampling adequacy for the analysis, KMO = 0.724 (‘good’ sample size). Barlett’s test of sphericity *x*^2^ (55) = 742.3, p <; 0.001, indicated that correlations between items were sufficiently large for PCA [15]. An initial analysis was performed to obtain eigenvalues for each component in the data. Four factors had eigenvalues above Kaiser’s criterion of 1 and in combination explained 76.2% of the variance (Table 3). Our results revealed 4 main factors instead of 3 main factors (categories) as classified in the original BQ. These can be described as: factor 1-snoring behaviour; factor 2-daytime fatigue; factor 3-sleepiness during driving; and factor 4-hypertension or obesity. Factors 1 and 4 in the Thai BQ corresponded to categories 1 and 3 in the original BQ. Factors 2 and 3 in combination represented category 2 in the original BQ.

**Table 3:** Factor structure of the Thai BQ

**Items**	**Factor loading**	**Eigenvalue**	**% Variance**
*Factor 1: Snoring behaviour*		3.90	35.44
Do you snore?	0.93		
Your snoring is?	0.74		
How often do you snore?	0.79		
Has your snoring ever bothered other people?	0.93		
Has anyone noticed that you quit breathing during your sleep? If yes, how frequently?	0.95		
*Factor 2: Sleepiness during driving*		2.03	18.49
Have you ever nodded off or fallen asleep while driving a vehicle?	0.98		
How often does this occur?	0.98		
*Factor 3: Daytime fatigue*		1.40	12.69
How often do you feel tired or fatigued after your sleep?	0.75		
During your waking time, do you feel tired, fatigue or not up to par?	0.79		
*Factor 4: Hypertension or obesity*		1.05	9.54
Do you have high blood pressure?	0.70		
Do you have BMI ≥ 30 kg/m^2^?	0.31		

### Reliability of the Thai BQ and the Thai ESS

The internal consistency of factor 1 of the Thai BQ was high (Cronbach’s alpha = 0.89). However, factor 3 which depicted daytime fatigue, the internal consistency was low (Cronbach’s alpha = 0.42) [15]. Since the fourth factor was summed into only 1 in total when calculating the risk for OSA, this factor was excluded from the calculations of internal consistency. The overall internal consistency of the Thai BQ was acceptable (Cronbach’s alpha = 0.77). The test-retest reliability (Cohen’s kappa coefficient) of the Thai BQ ranged from 0.66 to 0.98, (substantial to almost perfect agreement) [16]. Factor 3 had the lowest kappa value (Table 4). The internal consistency of the Thai ESS as evaluated by Cronbach’s alpha was 0.59 (95% CI = 0.45-0.70) and the test-retest reliability calculated using Cohen’s kappa coefficient was 0.81 (95% CI = 0.60-1.00).

**Table 4:** Evaluation of internal consistency and test-retest reliability of the factors of the Thai BQ

**Factors**	**Number of questions**	**Cronbach’s alpha (95% CI)**	**Kappa (95% CI)**
1. Snoring behaviour	5	0.89 (0.85-0.92)	0.88 (0.79-0.97)
2. Sleepiness during driving	2	0.78 (0.68-0.86)	0.81 (0.60-1.00)
3. Daytime fatigue	2	0.42 (0.31-0.61)	0.66 (0.43-0.89)
4. Hypertension or obesity	2	-	0.98 (0.94-1.00)
Total questionnaire	11	0.77 (0.69-0.83)	0.86 (0.74-0.98)

The agreement of the diagnosis of the high risk of OSA from the Thai BQ and the Thai ESS was also demonstrated by Cohen’s kappa coefficient. The results showed kappa values of 0.29 (95% CI = 0.10-0.48), which means that the agreement of the Thai BQ and the Thai ESS was fair, (Table 5) [16].

**Table 5:** Agreement between the Thai BQ and the Thai ESS

**Questionnaire**	**Thai BQ**		**Total**
**Thai ESS**	**Low risk**	**High risk**	
Low risk	72 (94.7%)	20 (83.3%)	92 (92%)
High risk	4 (5.3%)	4 (16.7%)	8 (8%)
Total	76 (100%)	24 (100%)	100 (100%)

Kappa value 0.29, 95% CI = 0.10-0.48.

## Discussion

The Thai BQ was developed by translation and adaptation of the original BQ, which allows assessment of the risk of OSA in general populations [9]. The preliminary validity and reliability of the Thai BQ was examined. The results of the study showed that the Thai BQ had good internal consistency in factor 1 (snoring behaviour) but not in factor 3 (daytime fatigue). Overall, the Thai BQ had good internal consistency and good test-retest reliability. The BQ allows the stroke caregiver to respond to objective questions relating to factors including snoring behaviour, i.e., the questionnaire rates the loudness and frequency of snoring. This was also shown in the study of Sagaspe et al., where the results of self-reported and bed-partner-reported BQ were compared: it was found that the bed-partner-reported BQ showed slightly better results. The authors concluded that this may be due to better identification of snoring or excessive daytime sleepiness by bed partners [17].

The prevalence of high risk for OSA identified from this study was very low (24%), although the study was conducted in a higher mean age group (mean age of 64 years) compared to other studies [1-4]. Twenty one patients (87.5%) were diagnosed as being at a high risk for OSA mainly by categories 1 and 3, 1 patient (4.2%) by categories 1 and 2, 1 patient by categories 2 and 3, and 1 patient by all 3 categories. This result was similar to other cross-sectional studies and a meta-analysis which showed that category 1 and category 3 are significant elements in the OSA population [13,18,19].

Due to the fact that stroke mostly occurs in middle-aged and elderly people, stroke patients usually have a higher energy expenditure while walking or performing daily life activities [20,21]. This can lead to tiredness and there is a high prevalence of fatigue in stroke patients, ranging from 16.1–58.1% [22,23]. In addition, stroke patients were found to have a high prevalence of sleep disorders (78%) such as insomnia or OSA [24]. Stroke patients should have both a high percentage of positive in category 2 and high risk for OSA identified from the ESS. Surprisingly, this study did not demonstrate this and the positive result in category 2 is low. There are some possible explanations for the low positive results in category 2 such as: 1) the questions in category 2 (factor 3) may not directly reflect daytime fatigue and the patients’ caregivers may not report fatigue because they considered fatigue as normal for stroke patients; 2) Thai elderly normally take a nap during the day so they feel less sleepy or fatigued during their waking time-the questions in factor 3 mainly focus on daytime fatigue or tiredness but do not involve sleepiness; lastly, 3) another possibility may relate to the question regarding sleepiness while driving, since stroke patients do not normally drive. Thus questions 8 and 9 in category 2 will be reported as 0: this was found in 93% of the answers. This was also observed in the study of Banhiran et al. which studied the validity and reliability of the Thai ESS in able-bodied participants. They found that participants responded to the question related to sleepiness in a car while stopped for a few minutes in the traffic as “no chance of dozing” because most participants do not drive [11].

The low reporting of sleepiness while driving may explain why 2 factors from factor analysis (daytime fatigue and sleepiness during driving) from category 2 are yielded instead of only 1. As the original BQ mainly aimed to assess the risk of OSA in the general population, the questions need to be modified before use in stroke patients to conform better to their daily lives.

There was only fair agreement between high risk of OSA diagnosed from the Thai BQ and the Thai ESS. This may be a result of the difference between the questionnaires, as a high risk of OSA was diagnosed using different criteria. The high risk of OSA according to the ESS mainly focusses on daytime sleepiness while for the BQ in this study the diagnosis relied on snoring behaviour and the diagnosis of hypertension or obesity. The internal consistency and test-retest reliability were lower in the present study compared to a previous study in Thailand. This may be due to differences between the respective study populations: the study by Banhiran et al., included able-bodied subjects with a lower average age [11].

As there was a discrepancy between the results of the Thai BQ and the Thai ESS for identifying the high risk group for OSA, 10 patients in the discrepancy group agreed to be investigated by polysomnography. Of the 10 patients, 1 was diagnosed as high risk by the Thai ESS and 9 were diagnosed as high risk by the Thai BQ. All 10 patients were diagnosed as OSA by polysomnography. Although this study shows a trend towards the Thai BQ in diagnosing OSA in stroke patients, further examination of the screening properties of the Thai BQ is required.

There are some limitations in the study. Patients with cardiopulmonary disease, aphasia or cognitive perceptual deficit were excluded. This exclusion may account for the lower prevalence of high risk OSA identified in this study population. Furthermore, mainly because of resource limitations, all participants were unable to be studied using overnight polysomnography so the predictive validity and the test properties of the Thai BQ could not be demonstrated. This study may provide baseline knowledge for further development of a modified version of the Thai BQ for the diagnosis of high risk OSA for stroke patients.

The next step is to focus on modification of the Thai BQ in category 2. The question regarding sleepiness while driving may need to be changed to a more appropriate question involving sleepiness during daily activities that are relevant to stroke patients. The appropriate cut off point for BMI should also be evaluated since the diagnosis of obesity in Asia and in western countries is different [25,26]. Finally, a study of the discriminative and predictive validity of the BQ in comparison with the gold standard, which is polysomnography, should be carried out.

## Conclusion

In conclusion, the Thai BQ has been developed. The study showed that the Thai BQ has an acceptable preliminary internal consistency and test-retest reliability. As factor analysis revealed 4 factors, in contrast to the 3 factors in the original study, modification of the Thai BQ is required. Comparison with the gold standard for the predictive validity of the Thai BQ is desirable before administration of the questionnaire in Thai stroke patients.

## Competing interests

The authors declare no conflict of interest.

## Authors’ contributions

JitS and NU contributed the study design, data collection, analysis, interpretation, manuscript preparation and revision. KS and JiaS helped with study design, analysis, interpretation, manuscript preparation and revision. All authors read and approved the final version of the manuscript.

## Supplementary Material

Additional file 1 Supplementary materials. Click here for file

Additional file 2 Berlin Questionnaire (used with permission from Professor Nikolaus C. Netzer). Click here for file

Additional file 3 Thai Berlin Questionnaire. Click here for file
