# Supplementary material for: Preliminary validity and reliability of a Thai Berlin questionnaire in stroke patients
Source: BMC Res Notes. 2014 Jun 9;7:348. doi: 10.1186/1756-0500-7-348 (PMC4060851; doi:10.1186/1756-0500-7-348)
Supplement: Additional file 1 — Supplementary materials. [file 1756-0500-7-348-S1.doc]

**Additional file 1: Supplementary Materials**

1. Epworth Sleepiness Scale (ESS). Available at [http://www.proqolid.org/instruments/epworth_sleepiness_scale_ess?fromSearch=yes&text=yes#subtabs-3](http://www.proqolid.org/instruments/epworth_sleepiness_scale_ess?fromSearch=yes&text=yes#subtabs-3) or <http://epworthsleepinessscale.com/1997-version-ess/>
2. Thai Epworth Sleepiness Scale. Banhiran W, Assanasen P, Nopmaneejumruslers C, Metheetrairut C: **Epworth sleepiness scale in obstructive sleep disordered breathing: the reliability and validity of the Thai version.** *Sleep Breath* 2011, **15:**571-7.
