# Supplementary material for: Preliminary validity and reliability of a Thai Berlin questionnaire in stroke patients
Source: BMC Res Notes. 2014 Jun 9;7:348. doi: 10.1186/1756-0500-7-348 (PMC4060851; doi:10.1186/1756-0500-7-348)
Supplement: Additional file 2 — Berlin Questionnaire (used with permission from Professor Nikolaus C. Netzer). [file 1756-0500-7-348-S2.pdf]

Berlin Questionnaire (for sleep apnea)

Scoring Berlin questionnaire

Adapted from: Table 2 from Netzer, et al., 1999. (Netzer NC, Stoohs RA, Netzer CM, Clark K, Strohl KP.
Using the Berlin Questionnaire to identify patients at risk for the sleep apnea syndrome.
Ann Intern Med. 1999 Oct 5;131(7):485-91).

The questionnaire consists of 3 categories related to the risk of having sleep apnea.

Patients can be classified into High Risk or Low Risk based on their responses to the individual items and their overall scores in the symptom categories.

Categories and scoring:
Category 1: items 1, 2, 3, 4, 5.
Item 1: if '**Yes**', assign **1 point**
Item 2: if '**c**' or '**d**' is the response, assign **1 point**
Item 3: if '**a**' or '**b**' is the response, assign **1 point**
Item 4: if '**a**' is the response, assign **1 point**
Item 5: if '**a**' or '**b**' is the response, assign **2 points**

Add points. Category 1 is positive if the total score is 2 or more points
Category 2: items 6, 7, 8 (item 9 should be noted separately).
Item 6: if '**a**' or '**b**' is the response, assign **1 point**
Item 7: if '**a**' or '**b**' is the response, assign **1 point**
Item 8: if '**a**' is the response, assign **1 point**

Add points. Category 2 is positive if the total score is 2 or more points
Category 3 is positive if the answer to item 10 is 'Yes' OR if the BMI of the patient is greater than 30kg/m2.
(BMI must be calculated. BMI is defined as weight (kg) divided by height (m) squared, i.e., kg/m2).

High Risk: if there are 2 or more Categories where the score is positive
Low Risk: if there is only 1 or no Categories where the score is positive

Additional question: item 9 should be noted separately.

BERLIN QUESTIONNAIRE

Height (m) ________ Weight (kg)________ Age______ Male / Female
Please choose the correct response to each question.

CATEGORY 1

1. **Do you snore?**
_ a. Yes
_ b. No
_ c. Don't know

If you snore:
2. **Your snoring is:**
_ a. Slightly louder than breathing
_ b. As loud as talking
_ c. Louder than talking
_ d. Very loud – can be heard in adjacent rooms

3. **How often do you snore**
_ a. Nearly every day
_ b. 3-4 times a week
_ c. 1-2 times a week
_ d. 1-2 times a month
_ e. Never or nearly never

4. **Has your snoring ever bothered other people?**
_ a. Yes
_ b. No
_ c. Don't Know

5. **Has anyone noticed that you quit breathing during your sleep?**

_ a. Nearly every day
_ b. 3-4 times a week
_ c. 1-2 times a week
_ d. 1-2 times a month
_ e. Never or nearly never

CATEGORY 2

6. **How often do you feel tired or fatigued after your sleep?**
_ a. Nearly every day
_ b. 3-4 times a week
_ c. 1-2 times a week
_ d. 1-2 times a month
_ e. Never or nearly never

7. **During your waking time, do you feel tired, fatigued or not up to par?**
_ a. Nearly every day
_ b. 3-4 times a week
_ c. 1-2 times a week
_ d. 1-2 times a month
_ e. Never or nearly never

8. **Have you ever nodded off or fallen asleep while driving a vehicle?**
_ a. Yes
_ b. No

If yes:
9. **How often does this occur?**
_ a. Nearly every day
_ b. 3-4 times a week
_ c. 1-2 times a week
_ d. 1-2 times a month
_ e. Never or nearly never

CATEGORY 3

10. **Do you have high blood pressure?**
_ Yes
_ No
_ Don't know
