# Supplementary material for: Preliminary validity and reliability of a Thai Berlin questionnaire in stroke patients
Source: BMC Res Notes. 2014 Jun 9;7:348. doi: 10.1186/1756-0500-7-348 (PMC4060851; doi:10.1186/1756-0500-7-348)
Supplement: Additional file 3 — Thai Berlin Questionnaire. [file 1756-0500-7-348-S3.pdf]

BERLIN QUESTIONNAIRE

ความสูง (ซม.)_____________ น้ำหนัก (กก.)_________ อายุ __________ ชาย / หญิง

โปรดเลือกคำตอบที่ดีที่สุดในแต่ละคำถาม.

หมวดที่ 1

1. **คุณนอนกรนหรือไม่** ?
   - ☐ a. ใช่
   - ☐ b. ไม่
   - ☐ C. ไม่ทราบ

ถ้าหากคุณกรน :

2. **การกรนของคุณ**
   - ☐ a. ดังกว่าเสียงหายใจ
   - ☐ b. ดังเท่ากับการพูดคุยเล็กน้อย
   - ☐ C. เสียงดังกว่าการพูดคุย
   - ☐ d. เสียงดังมาก – สามารถได้ยินถึงห้องติดกัน

3. **คุณกรนบ่อยแค่ไหน**
   - ☐ a. เกือบทุกวัน
   - ☐ b. 3 – 4 ครั้งต่อสัปดาห์
   - ☐ c. 1 – 2 ครั้งต่อสัปดาห์
   - ☐ d. 1 – 2 ครั้งต่อเดือน
   - ☐ e. ไม่เคยหรือแทบจะไม่เคย

4. **การกรนของคุณรบกวนผู้อื่นหรือไม่**
   - ☐ a. ใช่
   - ☐ b. ไม่ใช่
   - ☐ c. ไม่ทราบ

5. **มีใครสังเกตว่าคุณหยุดหายใจในช่วงการนอนหลับหรือไม่**
   - ☐ a. เกือบทุกวัน
   - ☐ b. 3 – 4 ครั้งต่อสัปดาห์
   - ☐ c. 1 – 2 ครั้งต่อสัปดาห์
   - ☐ d. 1 – 2 ครั้งต่อเดือน
   - ☐ e. ไม่เคยหรือแทบจะไม่เคย

หมวดที่ 2

6. **คุณรู้สึกเหนื่อยหรือล้าหลังการนอนบ่อยเพียงใด** ?
   - ☐ a. เกือบทุกวัน
   - ☐ b. 3 – 4 ครั้งต่อสัปดาห์
   - ☐ c. 1 – 2 ครั้งต่อสัปดาห์
   - ☐ d. 1 – 2 ครั้งต่อเดือน
   - ☐ e. ไม่เคยหรือแทบจะไม่เคย

7. **ในระหว่างที่คุณตื่น, คุณรู้สึกเหนื่อยล้าหรือรู้สึกไม่ดีเหมือนเดิมหรือไม่** ?
   - ☐ a. เกือบทุกวัน
   - ☐ b. 3 – 4 ครั้งต่อสัปดาห์
   - ☐ c. 1 – 2 ครั้งต่อสัปดาห์
   - ☐ d. 1 – 2 ครั้งต่อเดือน
   - ☐ e. ไม่เคยหรือแทบจะไม่เคย

8. **คุณเคยเผลองีบหรือหลับขณะขับขี่ยานพาหนะหรือไม่** ?
   - ☐ a. ใช่
   - ☐ b. ไม่

หากใช่ :

9. **เหตุการณ์นี้เกิดบ่อยเพียงใด** ?
   - ☐ a. เกือบทุกวัน
   - ☐ b. 3 – 4 ครั้งต่อสัปดาห์
   - ☐ c. 1 – 2 ครั้งต่อสัปดาห์
   - ☐ d. 1 – 2 ครั้งต่อเดือน
   - ☐ e. ไม่เคยหรือแทบจะไม่เคย

หมวดที่ 3

10. **คุณมีความดันโลหิตสูงหรือไม่** ?
    - ☐ a. ใช่
    - ☐ b. ไม่ใช่
    - ☐ c. ไม่ทราบ

เกณฑ์การให้คะแนน

หมวดที่ 1 ข้อ 1- 5

ข้อ 1 หากตอบ a ให้ 1 คะแนน

ข้อ 2 หากตอบ c หรือ d ให้ 1 คะแนน

ข้อ 3 หากตอบ a หรือ b ให้ 1 คะแนน

ข้อ 4 หากตอบ a ให้ 1 คะแนน

ข้อ 5 หากตอบ a หรือ b ให้ 2 คะแนน

การรวมคะแนน หมวดที่ 1 ถือเป็นบวกเมื่อคะแนนรวมมากกว่าหรือเท่ากับ 2 คะแนน

หมวดที่ 2 ข้อ 6-8 (ไม่รวมข้อ 9)

ข้อ 6 หากตอบ a หรือ b ให้ 1 คะแนน

ข้อ 7 หากตอบ a หรือ b ให้ 1 คะแนน

ข้อ 8 หากตอบ a ให้ 1 คะแนน

การรวมคะแนน หมวดที่ 2 ถือเป็นบวกเมื่อคะแนนรวมมากกว่าหรือเท่ากับ 2 คะแนน

หมวดที่ 3 **จะถือเป็นบวกเมื่อตอบ a ในข้อ 10 คือเป็นความดันโลหิตสูง หรือ มีค่าดัชนีมวลกายมากกว่า 30** kg/m^2

การแปลผล

หากได้ผลเป็น**บวกมากกว่าหรือเท่ากับ 2 หมวด**ถือว่า**มีความเสี่ยงต่อการเกิดภาวะหยุดหายใจขณะหลับสูง**

หากได้ผลเป็นบวก **1หมวดหรือไม่มีหมวดใดมีค่าเป็นบวกถือว่ามีความเสี่ยงต่อการเกิดภาวะหยุดหายใจขณะหลับต่ำ**
